# Case Report: a novel non-canonical splice site variant in COL4A5 in a patient with Alport syndrome

**DOI:** 10.3389/fmed.2026.1820315

**Published:** 2026-06-19

**Authors:** Xue Wang, Linlin Dong, Hui Qiu, Beibei Wang, Sanfeng Wang, Weiran Zhou

**Affiliations:** 1 Department of Pediatric Nephrology, Children’s Hospital Affiliated to Shandong University, Jinan, Shandong, China; 2 Department of Pediatric Nephrology, Jinan Children’s Hospital, Jinan, Shandong, China

**Keywords:** Alport syndrome, COL4A5, exon skipping, minigene splicing assay, non-canonical splice site variant

## Abstract

Alport syndrome (AS) is a genetically heterogeneous disorder caused by mutations in type IV collagen genes, clinically characterized by progressive renal function deterioration. Despite advances in genetic screening technologies, cases resulting from non-canonical splice site variants remain diagnostically challenging, frequently leading to missed diagnoses or delayed therapeutic intervention. Here, we report a pediatric patient with AS and his mother, both harboring a novel splicing variant in the COL4A5 gene. The proband presented with microscopic hematuria and was found to carry a hemizygous COL4A5 variant (NM_033380.3: c.3374-3T > G) through whole-exome sequencing. To determine the pathogenicity of this variant, *in vitro* functional validation was performed using a minigene splicing assay, which confirmed aberrant mRNA splicing leading to exon 38 skipping. These findings established this variant as the genetic etiology of AS in this family. Through the integration of whole-exome sequencing and minigene splicing analysis, this study identifies c.3374-3T > G as a novel pathogenic non-canonical splice site mutation in the COL4A5 gene. Our findings not only expand the mutational spectrum of Alport syndrome and provide critical insights for early diagnosis and clinical management of similar cases, but also underscore the necessity of functional validation for non-canonical splice site variants.

## Introduction

Alport syndrome (AS) is a hereditary glomerular basement membrane disorder caused by mutations in the COL4A3, COL4A4, and COL4A5 genes, which encode the α3, α4, and α5 chains of type IV collagen, respectively. Characterized by marked genetic heterogeneity, the disease is defined clinically by persistent hematuria and progressive renal function deterioration, often accompanied by sensorineural hearing loss and ocular abnormalities, with many patients progressing to end-stage renal disease (ESRD) during adulthood (1). However, in autosomal dominant (AD) Alport syndrome, affected individuals typically present with persistent hematuria without hearing loss, ocular abnormalities, or typical glomerular basement membrane (GBM) lamellation (2). Alport syndrome has traditionally been considered a rare genetic disorder. However, a recent study based on the Genome Aggregation Database (gnomAD), comprising over 122,000 subjects, revealed that 1 in 106 individuals (0.9%) harbor heterozygous variants in COL4A3 or COL4A4 that is predicted to be pathogenic and can cause AD Alport syndrome, and 1 in 2,320 carry pathogenic variants causing X-linked Alport syndrome (XLAS) (3). With advances in molecular diagnostic technologies, next-generation sequencing-based genetic testing has become a critical tool for definitive diagnosis of AS. In patients with typical clinical phenotypes, The detection rate of pathogenic variants in the COL4A3, COL4A4, and COL4A5 genes reaches approximately 90% (4).

However, the diagnostic yield is influenced by multiple factors, particularly for sequence variants located in non-canonical splice regions (e.g., intronic positions ± 3 to ± 5). Such variants pose significant challenges for pathogenicity interpretation and are frequently classified as “variants of uncertain significance,” potentially leading to missed or delayed diagnoses. Functional assays are often required to validate the pathogenicity of these variants. The minigene splicing assay serves as a classic method for *in vitro* validation of splicing variants, enabling robust assessment of variant effects on mRNA splicing when patient tissue RNA is unavailable, thereby providing crucial evidence for pathogenicity determination.

In the present study, we identified a previously unreported non-canonical splice site variant (NM_033380.3: c.3374-3T > G) in the COL4A5 gene within a family with clinically suspected Alport syndrome. Through minigene splicing assay, we confirmed that this variant causes exon 38 skipping, thereby elucidating its pathogenic mechanism. This report aims to establish the molecular diagnostic basis for this variant and underscore the importance of functional validation in interpreting non-canonical splice site variants, offering guidance for clinical genetic counseling and patient management.

## Case presentation

**FIGURE 1:**
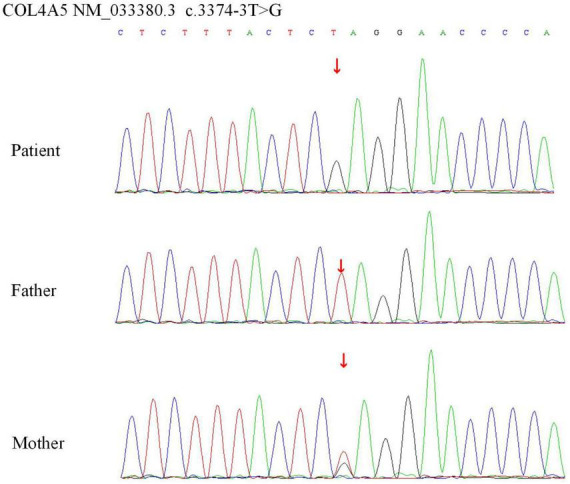
The proband’s family tree diagram, the arrow indicates the proband (solid black for affected hemizygous). The circle represents his mother (half-filled for carrier), and the squares represent the proband and his father.

The proband was an 11-year-and-9-month-old boy who presented with a one-month history of asymptomatic microscopic hematuria detected on routine urinalysis. One month prior to presentation, he was hospitalized for bronchitis at a local hospital, where urinalysis revealed positive occult blood test and red blood cells (RBCs) of 65.80/μL, without gross hematuria, proteinuria, or edema. On physical examination, his blood pressure was 100/70 mmHg; he exhibited normal growth and development, intact visual acuity bilaterally, and normal hearing. Cardiopulmonary examination yielded unremarkable findings. The abdomen was soft, with no percussion tenderness over the bilateral renal areas, negative shifting dullness, and no lower extremity edema. His past medical history was unremarkable, with no hearing impairment or ocular abnormalities. There was no known family history prior to the proband’s diagnosis. The proband’s mother, aged 37 years, is a carrier with intermittent microscopic hematuria (10.10–57.30/μL) without proteinuria. Renal function is normal (serum creatinine 58 umol/L, 122.5 ml/min/1.73m^2^). Audiometry revealed normal hearing. No ocular abnormalities were observed. The proband’s father showed no abnormalities in urinalysis, renal function tests, hearing, or ophthalmologic examinations. Unfortunately, we were unable to obtain relevant clinical information regarding the grandparents. Pedigree analysis suggested a possible X-linked dominant inheritance pattern (Figure 1). At our hospital, additional laboratory findings demonstrated RBCs of 72.30–274.40/μL on serial urinalyses, with UPCR 0.03–0.11 mg/mg and normal urinary renal early injury markers. Serum creatinine, complement levels, antinuclear antibodies (ANA), anti-streptolysin O (ASO), and complete blood count were all within normal limits. Renal ultrasonography revealed normal-sized kidneys with clear corticomedullary differentiation. Pure-tone audiometry was conducted, showing normal hearing thresholds bilaterally (≤25 dB HL at all frequencies tested: 125, 250, 500, 1000, 2000, 4000, and 8000 Hz). Slit-lamp examination was performed by an ophthalmologist, revealing no anterior lenticonus, corneal opacities, or perimacular flecks.

**FIGURE 2:**
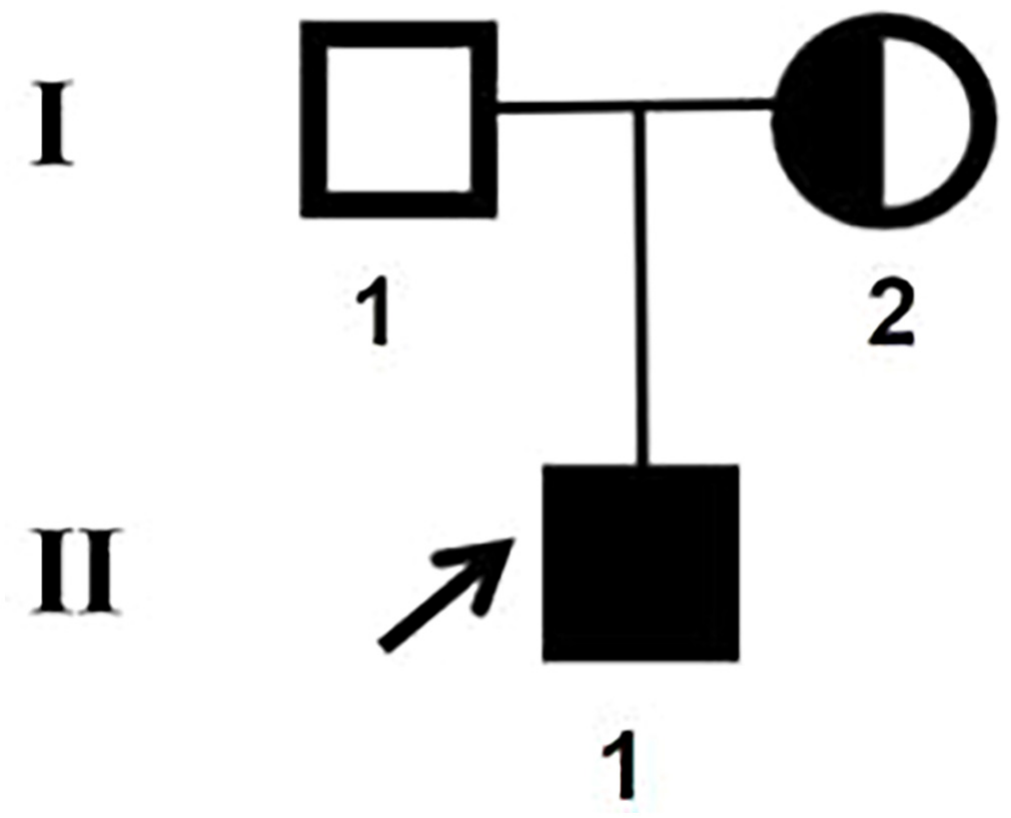
Sanger sequencing diagram. The mutation was verified by Sanger sequencing, which was inherited from the patient’s mother.

Genetic Analysis: With informed consent from the family, whole-exome sequencing (WES) was performed on peripheral blood from the proband. A hemizygous variant was identified in the COL4A5 gene: NM_033380.3: c.3374-3T > G (p.?) of maternal origin. This variant is located at the third nucleotide upstream of the 5′ end of exon 38, representing a non-canonical splice site variant. According to the American College of Medical Genetics and Genomics (ACMG) guidelines, this variant was initially classified as a variant of uncertain significance (VUS) (PM2_Supporting + PP3): PM2_Supporting-the variant was absent from normal population databases; PP3-spliceAI prediction score of 0.76, indicating an effect on splicing. No reports of this variant were identified in the literature, and no pathogenicity analysis was available in the ClinVar database. Segregation analysis confirmed that the father was wild-type at this locus, whereas the mother was heterozygous (Figure 2).

**FIGURE 3:**
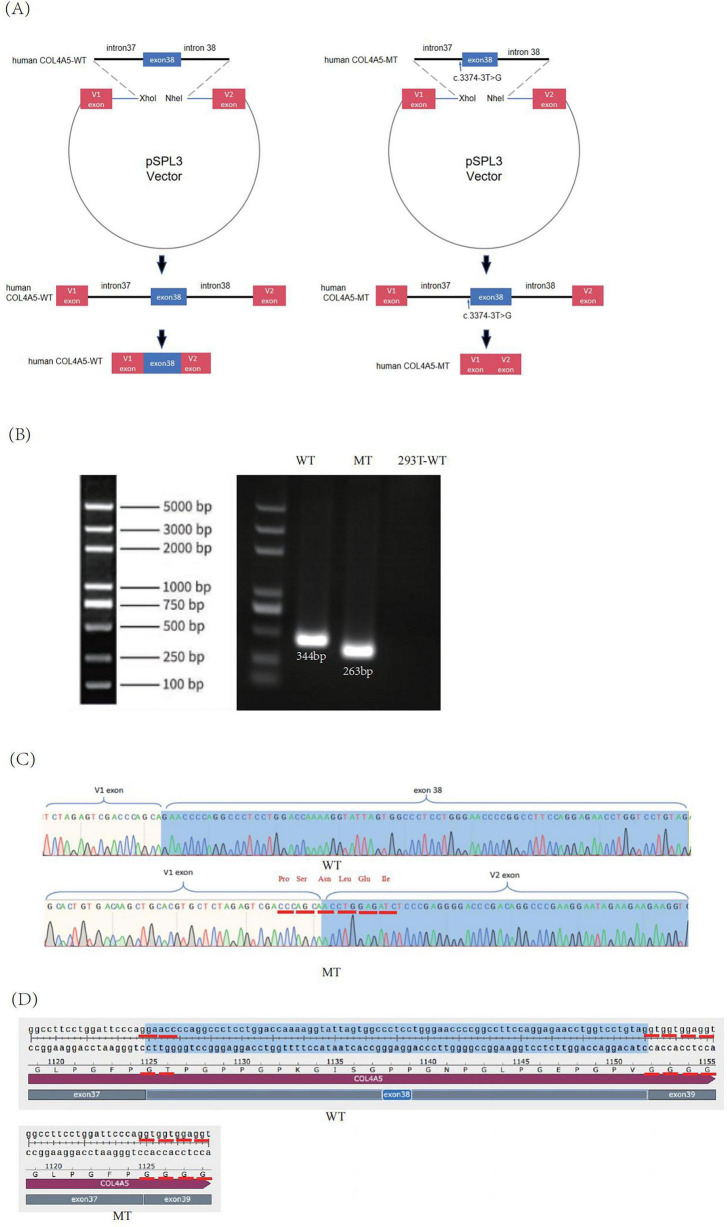
Minigene splicing assay transcript analysis. **(A)** Vector construction. Exon 38 of the COL4A5 gene (encompassing introns 37 and 38) was cloned into the pSPL3 vector, with predicted splicing outcomes illustrated. **(B)** Agarose gel electrophoresis demonstrating that the mutant type (MT) yielded a smaller amplicon compared with the wild type (WT). **(C)** Sanger sequencing confirmed exon 38 skipping. **(D)** Exon 38 skipping produces an 81-bp (27-amino-acid) in-frame deletion. The junctional codon GGT (glycine) is formed by the last base of exon 37 and the first two bases of exon 39, confirming the preservation of the reading frame.

Minigene Splicing Assay: Given the location of this variant in intron 37 of the COL4A5 gene, we hypothesized that it might affect pre-mRNA splicing, resulting in exon skipping or aberrant splicing. The pSPL3 vector is a eukaryotic expression vector specifically designed for minigene splicing assays. Its underlying principle utilizes the strong 5′ splice donor (SD) and 3′ splice acceptor (SA) sites derived from the HIV-Tat gene, which are intrinsic to the vector and situated between the V1 and V2 exons. A COL4A5 genomic DNA fragment (1994 bp) encompassing exon 38 with flanking intronic sequences, including the 3′ end of intron 37 (containing the c.3374-3T > G variant, 779bp) and the 5′ end of intron 38 (908bp), was amplified and cloned into the XhoI/NheI-linearized pSPL3 vector by seamless recombination using the GN-Trelief SoSoo Cloning Kit (Tsingke Biotechnology) (Figure 3A). The following primers were used for amplification: forward primer, 5′-TACGGGATCACCAGAATTCTGGAGCTCGAGggtgagcctggtctg cctgg-3′; reverse primer, 5′-TATTTCCAAATTGTTCTCTTAATT TGCTAGCctggttcacccttctgtccagct-3′ (uppercase letters indicate vector-derived sequences for recombination, and lowercase letters indicate target sequences). The PCR product was gel-purified and recombined with the linearized vector. Positive clones were screened by colony PCR and verified by Sanger sequencing. Wild-type (WT) and mutant (MT) constructs were subsequently transfected into 293T cells using Lipofectamine 3000. After 72 h, RNA was extracted, reverse-transcribed, and subjected to PCR amplification using vector-specific primers (V1 and V2) and then sequenced. Agarose gel electrophoresis demonstrated that the MT yielded a smaller amplicon compared with the WT (Figure 3B). Sanger sequencing confirmed that the splicing variant caused skipping of exon 38 (Figure 3C). These findings demonstrated that, in contrast to the wild-type, which produced normal transcripts containing exon 38, the c.3374-3T > G mutation generated aberrant transcripts with complete skipping of exon 38, causing an 81-bp/27-amino-acid in-frame deletion (Figure 3D), thereby confirming the deleterious effect of this variant on splicing function at the RNA level. Based on the ACMG guidelines, this variant is classified as Likely Pathogenic (PS3 + PM4 + PM2_Supporting): PS3: The minigene splicing assay demonstrated that this non-canonical splice site variant causes complete skipping of exon 38, resulting in an in-frame deletion of 27 amino acids; PM4: The deleted sequence removes nine consecutive Gly-X-Y repeat units within the collagenous triple-helical domain, directly disrupting the periodic structure essential for triple-helix formation; PM2_Supporting: The variant is absent (allele frequency = 0) from normal population.

The proband was diagnosed with XLAS based on clinical manifestations, family history, and molecular genetic evidence. Treatment was initiated with the angiotensin-converting enzyme inhibitor (ACEI) enalapril maleate (10 mg twice daily), combined with the traditional Chinese medicine Bailing Pian (1.8 g three times daily) as an empirical adjunctive therapy. After 2 years of follow-up, the patient’s urinary RBC count had declined to 1.44–12.20/μL, with undetectable urinary protein and microalbumin, normal renal function, and stable blood pressure. Hearing and ophthalmologic examinations remained normal. The mother currently exhibits isolated microscopic hematuria without pharmacological intervention, with preserved renal function on longitudinal monitoring.

## Discussion

Alport syndrome (AS) is a hereditary glomerular basement membrane disorder caused by mutations in the COL4A3, COL4A4, and COL4A5 genes. Although standard whole-exome sequencing (WES) and targeted next-generation sequencing (NGS) can detect approximately 82%–86% of pathogenic variants, certain genetic alterations (such as deep intronic variants, somatic mosaicism, and copy number variations) remain undetectable by these pproaches, leading to potential missed diagnoses and diagnostic delays (5). Literature reports indicate that nearly 90% of male patients with X-linked Alport syndrome (XLAS) develop chronic kidney disease (CKD) before the age of 40 years, with subsequent progression to end-stage renal disease (ESRD) (6). A prospective study in pediatric patients with AS demonstrated that children treated with ramipril at very early disease stages (urinary albumin-to-creatinine ratio (UACR) <300 mg/g or microhematuria) exhibited diminished the slope of albuminuria progression (7). Furthermore, studies have shown that patients receiving angiotensin-converting enzyme (ACE) inhibitor therapy experience prolonged time to renal failure, with greater benefits observed when treatment is initiated at earlier disease stages (8). These findings underscore the critical importance of reducing the rate of missed diagnoses and enabling early diagnosis and treatment to improve prognosis.

Significant genotype–phenotype correlations have been established in male patients with XLAS. Approximately 15% of pathogenic mutations in XLAS involve splice site variants (9). Splice mutations represent an important pathogenic mechanism, with specific sites and types profoundly influencing disease progression and prognosis. Studies have demonstrated that, compared with missense mutations, splice mutations typically result in earlier onset of end-stage renal disease (ESRD), with a mean age at onset of approximately 28 years and a 70% risk of progression to ESRD by age 30 years (10, 11). However, heterogeneity within splice mutations is equally critical: patients with truncating versus non-truncating mutations exhibit median ages at ESRD of 20 versus 29 years, respectively (12). Additionally, significant differences exist between patients with in-frame versus out-of-frame splice variants; those with in-frame variants demonstrate a median time to renal failure of 28 versus 23 years for those with out-of-frame variants (13). Synthesis of these findings with our patient’s genetic profile reveals that our proband carries an in-frame deletion, placing him statistically at high risk for ESRD in his late 20s. This prognostic insight has direct clinical implications, providing a robust rationale for early initiation of angiotensin-converting enzyme inhibitor (ACEI) therapy at age 11. Preemptive ACEI administration represents a critical intervention to modify disease trajectory, delay renal function deterioration, and potentially prolong the time to ESRD onset. Therefore, precise functional analysis and classification of splice variants are indispensable for assessing renal prognosis and providing genetic counseling.

Previous studies have established that exon 38 skipping in the COL4A5 gene represents a definitive pathogenic mechanism in X-linked Alport syndrome (XLAS); however, reported cases have predominantly resulted from mutations at canonical splice sites. Notably, although the reported variants differ in their genomic positions, they all lead to an identical molecular consequence: in-frame skipping of exon 38, resulting in the same aberrant α5 (IV) chain. Given that these variants produce the same aberrant transcript and are thus expected to confer similar pathogenicity, we compared the phenotypic spectrum between our proband and the reported cases. Patients described by Netzer et al. (NM_033380.3:c.3454+1G > C) (14) and Hertz et al. (NM_033380.3:c.3454+1G > T) (15) both presented with hearing impairment and adolescent-onset Alport syndrome progressing to end-stage renal disease, with or without ocular involvement. However, Daga et al. (16) reported a 4-year-old boy who developed persistent gross hematuria following a urinary tract infection, with proteinuria detected on a single occasion; both ophthalmologic and hearing assessments were normal. Targeted genetic testing identified a COL4A5 intronic variant (NM_033380.3:c.3454+2T > C) that similarly caused exon 38 skipping. In our case, the patient was an 11-year-old boy presenting with microscopic hematuria, normal renal function, and no hearing or ocular impairment. These observations suggest that extra-renal manifestations of exon 38 skipping may be age-dependent and evolve over time. Collectively, these comparisons reinforce that exon 38 skipping exerts a consistent pathogenic effect on the glomerular basement membrane (GBM), while phenotypic expressivity may vary with age and genetic background. Long-term clinical follow-up will be critical to assess potential progression.

All aforementioned reports involved canonical splice site variants. Nevertheless, aberrant splicing of the COL4A5 gene can also result from non-canonical mechanisms, including alterations in deep intronic regions or activation of cryptic splice sites within exons (9). Unlike previously reported canonical splice variants, the c.3374-3T > G variant identified in the present study is located at the −3 position of the splice acceptor site in intron 37, representing a non-canonical splice site variant. This variant has not been previously reported, and its pathogenicity interpretation presents greater challenges, with a propensity for classification as a variant of uncertain significance and consequent missed clinical diagnosis.

Our functional validation demonstrates that pathogenic variants at non-canonical splice sites can similarly cause skipping of critical exon 38. As previously reported, exon 38 encodes 27 amino acids (81 bp) within the collagenous triple-helical domain of the α5 (IV) chain, comprising nine consecutive Gly-X-Y repeat units (17). Although exon 38 skipping results in an in-frame deletion without frameshift, it directly disrupts the periodic structure essential for triple-helix formation. Theoretical models suggest that such mid-domain deletions may introduce conformational entropy barriers, interfering with the “zipper-like” folding nucleation process initiated from the C-terminal NC1 domain, thereby compromising proper assembly and stability of the α3α4α5 (IV) heterotrimer (18, 19), providing a structural basis for understanding its pathogenicity. In the present case, the proband presented with microscopic hematuria at 11 years of age. Although proteinuria had not yet developed, the resulting deletion of exon 38 at the transcript level, in conjunction with previous reports, predicts substantial disruption of glomerular basement membrane structural integrity, necessitating close monitoring of renal function progression. Furthermore, this case underscores the necessity of experimental validation for non-canonical splice site variants in patients with high clinical suspicion of AS, thereby preventing missed diagnoses and delayed treatment.

Genetic testing has emerged as a first-line diagnostic tool for AS owing to its noninvasive nature and high detection rate, providing critical information for genetic counseling and family screening. Current international consensus recommends that genetic testing should precede renal biopsy in patients with clinical suspicion of AS; when a definitive pathogenic variant is identified, renal biopsy can typically be avoided (20, 21). However, for novel non-canonical splice variants such as that identified in the present study, pathogenicity confirmation often requires functional experimental support. Although RNA sequencing using patient-derived urinary cells or skin fibroblasts represents an ideal *in vivo* validation method, practical limitations frequently arise from RNA degradation, low cell yield, or technical complexity (22, 23). The minigene splicing assay, as a well-established *in vitro* functional validation system, offers a reliable and efficient solution in this context (24). The present study directly employed this strategy to successfully elucidate the functional impact of the variant in the absence of accessible patient tissue, thereby providing crucial evidence for molecular diagnosis and early risk stratification.

## Conclusion

Through molecular genetic analysis of a family with AS, this study reports a novel non-canonical splice site variant (c.3374-3T > G) in the COL4A5 gene and demonstrates its pathogenicity through *in vitro* minigene splicing assay, thereby expanding the mutational spectrum of AS. Early identification of this variant enabled the proband to receive standardized intervention prior to renal injury, improving long-term prognosis. This study is limited by the lack of *in vivo* RNA validation and its single-family design. The genotype-phenotype correlation and long-term prognosis of this variant remain to be defined through continued longitudinal follow-up of this family and systematic compilation of future cases reported in the literature. The functional validation strategy established in this study provides a reproducible model for the diagnosis of similar splice variants.

## Data Availability

The novel variant NM_033380.3:c.3374-3T>G has been submitted to ClinVar under accession number VCV004850880.1 and is publicly available at https://www.ncbi.nlm.nih.gov/clinvar/variation/VCV004850880.1. All other relevant data are available within the article and its supplementary material.
